# Gastrostomie percutanée endoscopique: une approche innovante pour la nutrition entérale

**DOI:** 10.11604/pamj.2026.53.25.47935

**Published:** 2026-01-19

**Authors:** Sara Darhoua, Fatima-Zahra Belabbes, Hanane Delsa, Anass Nadi, Omar Bahlaoui, Wafaa Khannoussi, Imane Ben El Barhdadi

**Affiliations:** 1 Département de Gastro-entérologie et de Proctologie, Hôpital Universitaire International Cheikh Khalifa, Casablanca, Maroc,; 2 Département de Gastro-entérologie et de Proctologie, Hôpital Mohammed VI Bouskoura, Université des Sciences et de la Santé Mohammed VI (UM6SS), Casablanca, Maroc

**Keywords:** Nutrition entérale, gastrostomie endoscopique percutanée, complications, Enteral nutrition, percutaneous endoscopic gastrostomy, complications

## Abstract

La gastrostomie percutanée endoscopique (GPE) permet un accès nutritionnel entéral durable. Cette étude vise à évaluer la faisabilité, les indications, le taux de succès et les complications liées à la GPE. Il s'agit d'une étude rétrospective descriptive menée sur 93 patients entre 2020 et 2023 à l'Hôpital Universitaire Cheikh Khalifa. Les données épidémiologiques, cliniques et biologiques ont été analysées. Une régression logistique multivariée a été réalisée pour identifier les facteurs associés aux complications. L'âge moyen était de 70 ± 14 ans; 72% d'hommes. Les principales indications étaient neurologiques (76%) et l'oto-rhino-laryngologie (ORL) (24%). Le taux de succès était de 100%. Des complications mineures ont été observées chez 7,5%: infection péristomiale (2,1%), obstruction (3,2%), fuite (2,1%). Les facteurs prédictifs significatifs étaient l'hospitalisation en soins intensifs (p < 0,0001), l'hypoalbuminémie < 30 g/L (ORa: 15,5; IC 95%: 1,77-135,6; p = 0,0042) et l'indication neurologique (ORa: 6,1; IC 95%: 1,11-33,55; p = 0,0335). La GPE est une technique sûre et efficace pour l'accès nutritionnel prolongé, avec un faible taux de complications lorsque les mesures préventives sont respectées.

## Abstract (en)

Percutaneous endoscopic gastrostomy (PEG) provides long-term enteral nutritional access. This study aims to evaluate the feasibility, indications, success rates and complications associated with PEG. A retrospective descriptive study was conducted on 93 patients between 2020 and 2023 at Sheikh Khalifa University Hospital. Epidemiological, clinical and biological data were analysed. Multivariate logistic regression was performed to identify factors associated with complications. The mean age was 70 ± 14 years; 72% were men. The main indications were neurological (76%) and ENT (24%). The success rate was 100%. Minor complications were observed in 7.5%: peristomal infection (2.1%), obstruction (3.2%), leakage (2.1%). Significant predictive factors were intensive care unit admission (p < 0.0001), hypoalbuminaemia < 30 g/L (ORa: 15.5; 95% CI: 1.77-135.6; p = 0.0042) and neurological indication (ORa: 6.1; 95% CI: 1.11-33.55; p = 0.0335). PEG is a safe and effective technique for prolonged nutritional access, with a low complication rate when preventive measures are followed.

## Introduction

La nutrition entérale (NE) présente plusieurs avantages par rapport à la nutrition parentérale, car elle permet de préserver l'architecture des muqueuses, ce qui réduit le risque d'inflammation, de fuite intestinale et d'infections dues à la pathogénicité du microbiote intestinal [1]. Elle permet également de préserver la fonction immunitaire hépatique et pulmonaire. L'alimentation entérale a permis de réduire la durée du séjour à l'hôpital et de minimiser la nécessité d'une ventilation mécanique chez ces patients [2].

La nutrition entérale et la mise en décharge du tube digestif sont les deux circonstances nécessitant la mise en place d'une sonde gastrique. Au stade aigu, on utilise généralement une sonde nasogastrique (SNG), efficace et de mise en place simple au lit du patient. La GPE a été décrite pour la première fois en 1980. Cependant, la SNG présente des inconvénients lorsque son usage se prolonge sur plusieurs semaines, donnant des irritations locales, un reflux, une obstruction du fait du faible calibre, voire un handicap esthétique et social [3]. Son utilisation doit donc être limitée dans le temps. La gastrostomie est alors l'alternative, créant un abord gastrique pérenne par voie transpariétale directe. La gastrostomie doit être proposée lorsque la nutrition gastrique doit se prolonger de 4 à 6 semaines [4]. Elle convient aux patients dont la fonction intestinale est normale, mais dont la déglutition et la nutrition orale sont déficientes. Les indications de la GPE sont compatibles avec la gastrostomie chirurgicale, comme les accidents vasculaires cérébraux, les tumeurs oropharyngées ainsi que les troubles de la déglutition. Depuis sa description par Ponsky *et al*. en 1980, la GPE a connu un essor considérable et est devenue la méthode de référence pour l'accès entéral à long terme [5]. Il s'agit d'un acte simple, rapide, réalisable par tout endoscopiste, mais nécessitant une procédure très rigoureuse.

Des complications peuvent survenir avec la mise en place d'une GPE. Les complications possibles sont des douleurs, une fuite du contenu gastrique. Plus rarement, il peut survenir une infection du site de GPE, une hémorragie ou une perforation [6]. Le but de notre travail est d'analyser les indications, ainsi que l'évolution des malades ayant bénéficié d'une GPE, et de déterminer les facteurs prédictifs de survenue de complications.

## Méthodes

**Conception et cadre de l'étude:** étude descriptive rétrospective de patients pris en charge au service de gastro-entérologie de l'Hôpital Universitaire Cheikh Khalifa de Casablanca. Notre travail a été étalé sur une durée de 3 ans entre 2020 et 2023. Ont été inclus tous les patients chez qui l'indication de la mise en place d'une gastrostomie percutanée endoscopique a été retenue. Donc le nombre de patients ayant bénéficié de la GPE était de 93.

### Participants et critères d'admissibilité

**Critères d'inclusion:** patients ayant des difficultés à avaler pendant au moins 1 mois ou qui ont une espérance de vie supérieure à 3 mois. Les patients ne présentant aucune contre-indication pour la nutrition entérale. Le geste a été fait après l'obtention d'un consentement éclairé de la part des membres de la famille.

**Critères d'exclusion:** les femmes enceintes et les patients présentant des signes de dysfonction organique ou de détérioration clinique grave.

**Collecte de données:** le recueil des données suivantes a été réalisé à partir du registre d'endoscopie du service et à l'aide du dossier informatisé à travers le logiciel DXCARE dans notre structure hospitalière: l'âge et le sexe des patients, les indications de pose de la sonde de GPE, le succès de pose de la sonde de GPE, les complications, les causes éventuelles de changement de sonde et ATCDs, le taux d'albumine. Les patients ont été suivis dans la première semaine, à 3 mois et à 6 mois après la mise en place de la GPE.

**Figure 1:**
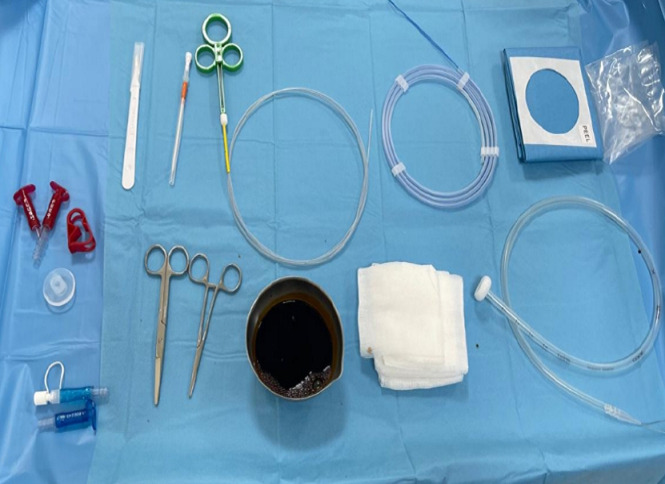
kit de la gastrostomie percutanée endoscopique

**Matériels utilisés:** le matériel nécessaire à la réalisation de cette technique était: une fibroscopie œsophage-gastro-duodénale (FOGD) et un kit complet de GPE stérile. Les kits de GPE utilisés comportent une sonde de gastrotomie, un trocart de ponction avec manchon amovible, un fil guide, un bouchon raccord adaptable à la sonde, un champ stérile, une anse, des ciseaux, une porte-aiguille et des compresses stériles (Figure 1).

**Méthode d'évaluation:** dans le but de déterminer les facteurs prédictifs de survenue de complications, nous avons analysé 6 variables: l'âge avancé ≥70 ans, le sexe masculin, une hospitalisation prolongée en unité de soins intensifs, l'indication de la GPE, la dénutrition par un faible taux d'albumine (<30 g/l) et les comorbidités du patient telles que diabète, maladie coronarienne, infarctus du myocarde récent (<6 mois), bronchopneumopathie obstructive chronique, infection concomitante (infection des voies urinaires, pneumonie). Le succès a été évalué six semaines après le début de l'alimentation et les changements dans l'état nutritionnel pendant cette période. L'échec du traitement a été défini comme le défaut de localisation de la sonde de GPE ou son déplacement récurrent.

**Analyse de données:** les logiciels utilisés pour les analyses des données sont le logiciel SPSS (Statistical Package for the Social Sciences) et Excel. Le test chi^2^ et le test de Fisher étaient utilisés dans l'analyse statistique. Le seuil de significativité était (p < 0,05).

## Résultats

**Figure 2:**
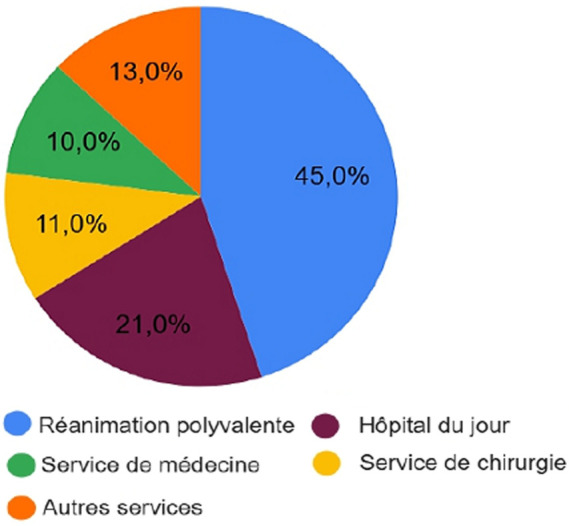
répartition des patients en fonction du service d'origine

**Caractéristiques des patients:** l'indication de la GPE a été retenue chez 93 patients; l'âge moyen des patients était de 70 ans, avec des extrêmes de 12 et 90 ans. La tranche d'âge la plus représentative était de 71 à 90 ans (58%, n=54), 32% (n= 30) de nos patients entre 51 et 70 ans, 6% (n=5) entre 31 et 50 ans, et 4% (n=4). Nous avons constaté que les hommes étaient majoritaires, ils représentaient 72%, alors que les femmes ne représentaient que 28%. La majorité des patients, 45% du total, étaient hospitalisés dans le service de réanimation polyvalente, 21% dans l'hôpital du jour, 11% dans le service de chirurgie, 10% dans le service de médecine, et les 13% restants se répartissaient respectivement dans chacun des services d'unité de soins intensifs, de pédiatrie et de trauma center (Figure 2).

**Figure 3:**
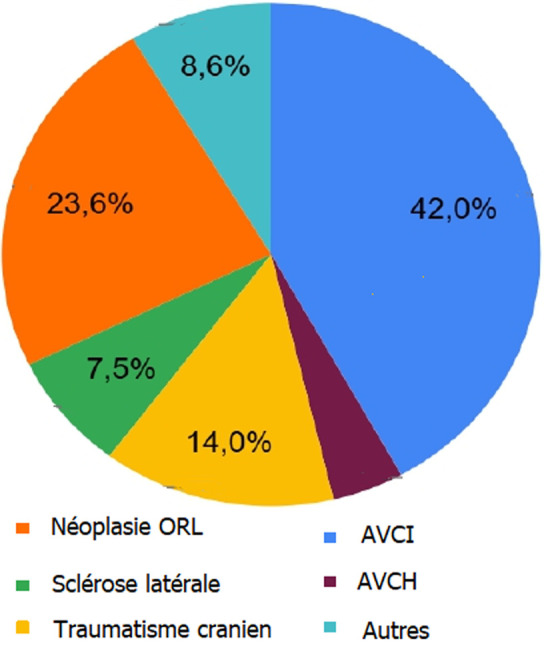
indications de pose de la gastrostomie percutanée endoscopique

**Indications:** dans notre série, les indications de la GPE sont surtout représentées par les pathologies neurologiques et les cancers ORL qui présentent respectivement 76% (n=71) et 23,6% (n=22). Les résultats sont résumés dans le Tableau 1 et représentés graphiquement dans la Figure 3. Il faut noter que l'association d'un ou de plusieurs signes fonctionnels à ces pathologies a permis de prendre la décision de réaliser la gastrostomie percutanée endoscopique chez ces malades.

**Tableau 1:** indications de la gastrostomie percutanée endoscopique

Indications	Pourcentage % (n)
Accident vasculaire cérébral ischémique	42% (n=39)
Accidents vasculaires cérébraux hémorragiques	4,3% (n=4)
Traumatismes crâniens	14 % (n=13)
Sclérose latérale amyotrophique	7,5 % (n=7)
Maladie de Parkinson	4,3% (n=4 )
Démence	2,1% (n=2)
Cancer du larynx	50% (n=11)
Cancer de la cavité buccale	32% (n=7)
Cancer du cavum	18% (n=4)

**Complications:** le taux de succès était de 100%, avec l'absence de complications immédiates. Des complications mineures à distance (plus de 1 mois) ont été observées chez 7,5% (n=7) des patients, une infection péristomiale dans 2,1% (n=2) ayant nécessité l'ablation de la sonde et une antibiothérapie adaptée, un blocage de la sonde dans 3,2% (n=3) des patients et des fuites de liquide dans 2,1% (n=2) des patients.

**Evolution:** 7,5% des patients (n=7), ont bénéficié d'une ablation de sonde due à une amélioration des signes cliniques (absence de trouble de déglutition) et de l'état nutritionnel, dont 4 malades étaient hospitalisés pour traumatisme crânien, 3 pour accident vasculaire cérébral. De plus, 21,7% des patients (n=20) ont bénéficié d'un changement de sonde, dont la raison principale, chez 10% (n=9) a été une usure du matériel. Les autres causes sont les suivantes: 6,4% d'arrachements de sonde, chez les patients présentant un état d'agitation (n= 6), 3,2% due à une infection péristomiale (n=3), 2,1% de fuites de liquide (n=2). Il y a eu une légère augmentation des taux d'albumine sérique, qui sont passés de moins de 30 mg avant la pose de la GPE à 35 mg au cours du suivi. Il faut noter que 8,6% (n=8) ont maintenu une alimentation entérale par la GPE; le reste des malades (n=58) est perdu de vue après 9 mois de suivi, ce qui peut être considéré comme une limite de l'étude. La durée d'utilisation était variable selon les indications, avec une durée moyenne de 9 mois et des extrêmes allant de 4 mois chez les patients atteints de traumatisme crânien et d'accident vasculaire cérébral à 12 mois pour les néoplasies ORL. Il faut noter que chez les patients atteints de sclérose latérale amyotrophique, la gastrostomie percutanée endoscopique est maintenue, vu l'installation définitive des troubles de la déglutition.

**Facteurs associés aux complications:** parmi les 93 patients ayant bénéficié d'une gastrostomie per-endoscopique (GPE), des complications à distance ont été observées chez 7,5% des cas. L'analyse des facteurs prédictifs a révélé une association significative entre la survenue de complications et plusieurs variables cliniques. L'hospitalisation en soins intensifs était fortement associée aux complications (p < 0,0001). De même, une hypoalbuminémie <30 g/L représentait un facteur de risque significatif (OR = 15,5; IC95% [1,77-135,6]; p = 0,0042). L'indication neurologique pour la pose de la GPE s'est également révélée être un prédicteur indépendant (OR = 6,1; IC95% [1,11-33,55]; p = 0,0335). En revanche, ni l'âge ≥70 ans ni le sexe masculin ne présentaient de lien statistiquement significatif avec la survenue de complications dans cette série (Tableau 2).

**Tableau 2:** facteurs prédictifs de complications après mise en place de la gastrostomie percutanée endoscopique

Facteurs prédictifs	OR 1	p-value
Âge ≥ 70 ans	-	0,5876
Sexe masculin	2,46	0,6687
Hospitalisation en unité de soins intensifs	-	<0,0001
Hypoalbuminémie (<30 g/L)	15,5	0,0042
Indication neurologique	6,1	0,0335
1 OR = rapport de cotes	-	-

## Discussion

Dans notre étude, les indications et les complications possibles de la GPE ont été examinées. L'indication pour le placement de la GPE était neurologique, particulièrement l'AVC dans 37%. La complication cardinale était l'infection péristomiale. Cette étude a observé un taux de réussite de 95,9%. La technique « Pull », ayant fait l'objet de multiples améliorations, est devenue simple, rapide, fiable, bien tolérée et réalisable par tout endoscopiste, mais nécessite une procédure très rigoureuse [6]. Il n'existe pas de consensus concernant les indications de la GPE [7], car il faut juger de l'impact de la gastrostomie sur l'amélioration de la qualité de vie du patient, sur son statut fonctionnel et sur sa survie, en évaluant le bénéfice-risque du geste [8]. Parmi les indications de la pose d'une GPE, nous retrouverons [9,10], trouble de la déglutition, d'origine neurologique (AVC, coma prolongé, sclérose en plaques, maladie de Parkinson, sclérose latérale amyotrophique…), ORL, traumatique, affection neuromusculaire, pneumopathies d'inhalation (par fausse route, perte d'autonomie du sujet âgé); dysphagie d'origine œsophagienne (sténoses bénignes ou malignes en cas d'échec des techniques endoscopiques palliatives), ORL, séquelles chirurgicales ou radiques; troubles alimentaires comme l'anorexie et la dépression et trouble de l'absorption en cas de maladie de Crohn, sclérodermie, de grêle radique ou court. Si la GPE est indiquée dans plusieurs pathologies, cette technique est pratiquée dans le service de réanimation de l'Hôpital Universitaire Cheikh Khalifa au profit des patients qui souffraient essentiellement de pathologies neurologiques dans 37% des cas, notamment des accidents vasculaires cérébraux ischémiques; cela est compatible avec des études antérieures (Tableau 3) [11].

**Tableau 3:** indications de la gastrostomie percutanée endoscopique selon les études

Indications	Rostoker, (France), 2002	Vanis, (Bosnie), 2012	Mekhail, (EU), 2001	El Anguoud, (Maroc), 2019	Notre étude
AVC	57%	20%	57%	17%	46%
Néoplasie ORL	4%	44%	3%	29%	24%
Traumatisme crânien		16%	12%	7%	14%
Démence	22%	1.3%	-	-	2%

Il est nécessaire de respecter certaines contre-indications afin de réduire les échecs de pose et la morbidité. La recherche dans ce domaine a montré qu'il existe deux types de contre-indications. La contre-indication la plus fréquente semble être la «mauvaise indication» que tout médecin devrait pouvoir détecter [12]. D'après l'étude des cas cliniques du service de l'hôpital Cheikh Khalifa, nous avons constaté que les patients étaient favorables à la GPE puisqu'ils n'ont pas présenté de contre-indications. Les complications des gastrostomies percutanées, à distance de la pose, sont les plus fréquentes des complications locales touchant l'orifice (fuites, suppuration, infection, bourgeon charnu) et des incidents matériels (obstruction, migration). Ces complications sont généralement bénignes et ce n'est qu'exceptionnellement que peuvent survenir des complications graves comme une fasciite nécrosante [13]. Cependant, on peut classer les complications en catégories: les complications majeures. Leur incidence est de 0 à 5% en fonction des études [14]. Elles comprennent la péritonite, la pneumopathie d'inhalation, la fasciite nécrosante, la nécrose pariétale, l'hémorragie digestive, la déchirure œsophagienne, la perforation gastrique, l'hémorragie intra-abdominale, la fistule colo-cutanée [15]. Les complications mineures sont fréquentes et ne gênent pas la poursuite de l'alimentation; leur incidence est comprise entre 13 et 43% dans les différents essais retrouvés dans la littérature, l'infection péristomiale étant la plus fréquente [16], comme ce fut le cas dans notre série.

Erdil *et al*. [17], dans une étude sur 85 en 2004, ont signalé 14 complications majeures dans moins de 30 jours chez 10 patients et 18 complications à long terme chez 12 d'entre eux. D'autres complications peuvent survenir, à savoir : dysfonction du tube (bouchage, plicature, déplacement, chute). Le nombre réduit de complications au service peut être justifié soit par l'effectif limité de patients au service, soit par l'attitude rigoureuse de l'équipe durant la pratique de la GPE depuis la préparation du patient jusqu'à l'administration de soins, soit par les deux considérations. Les patients dénutris présentent un risque trois fois plus élevé de complications précoces, généralement des abcès de paroi et des pneumopathies d'inhalation, par rapport aux patients non dénutris, selon l'étude de McClave *et al*. [16]. Une durée d'hospitalisation dépassant 10 jours avant la pose de la GPE était également associée à un risque accru de complications.

Dans notre étude, plusieurs facteurs ont été identifiés comme prédictifs de complications après la mise en place d'une GPE, notamment l'hospitalisation en soins intensifs, une hypoalbuminémie (<30 g/L) et une indication neurologique. Ces résultats sont en concordance avec les données de la littérature, qui ont également souligné l'importance de l'état clinique préalable des patients dans la survenue de complications post-procédure. L'association entre l'hospitalisation en soins intensifs et les complications est bien documentée. De nombreuses études montrent que les patients en soins intensifs, souvent plus fragiles et présentant des comorbidités multiples, ont un risque accru de complications après une GPE. De plus, notre constat d'une hypoalbuminémie significativement associée aux complications rejoint les conclusions de plusieurs études qui mettent en évidence le rôle crucial de la nutrition dans la cicatrisation et la gestion post-opératoire des patients [18]. En particulier, un faible taux d'albumine est souvent utilisé comme indicateur de dénutrition sévère, un facteur de risque bien reconnu dans le contexte des procédures invasives. Quant à l'indication neurologique, bien qu'elle soit moins fréquemment citée dans la littérature, plusieurs travaux confirment que les troubles neurologiques, en raison de la dépendance accrue et des complications associées, peuvent augmenter le risque de complications après des interventions comme la GPE. Cependant, le sexe masculin et l'âge ≥ 70 ans n'ont pas montré de lien significatif avec les complications dans notre étude, ce qui contraste avec certaines recherches où ces facteurs sont souvent associés à un risque plus élevé de complications post-opératoires [19].

Les taux de mortalité précoce (avant la fin du premier mois) les plus souvent rapportés après la pose de GPE varient entre 20 et 24% et sont rarement directement liés au geste [17]. Plusieurs facteurs de risque associés à un risque accru de décès précoce ont été identifiés, notamment l'âge avancé, le sexe masculin, la présence de diabète et la pathologie sous-jacente justifiant la pose de la gastrostomie. Erdil *et al*. ont souligné que les épisodes d'inhalation avant la pause étaient des prédicteurs de la mortalité à 30 jours. Dans notre étude, la satisfaction des proches a été obtenue. Dans une question ouverte visant à déterminer l'attitude à l'égard de la mise en place de la GPE, il a été constaté qu'elle était confortable, avec un niveau d'acceptation élevé par rapport à la sonde nasogastrique.

**Limites de l'étude:** la petite taille des échantillons et les taux faibles d'événements ne sont pas les seules limites. En effet, nous partageons les limites typiques des études rétrospectives. La catégorisation des symptômes, des dates, des évènements de façon rétrospective a pu être particulièrement difficile.

## Conclusion

La GPE est une technique sûre et efficace pour la nutrition entérale à long terme. Les complications restent rares et évitables par une sélection rigoureuse des patients et une prise en charge standardisée.

### Etat des connaissances sur le sujet

- La GPE est la méthode de référence pour la nutrition entérale prolongée;
- La technique « Pull » est la plus courante et la plus simple à réaliser;
- Les complications locales sont les plus fréquentes mais souvent mineures.

### Contribution de notre étude à la connaissance

- L'hypoalbuminémie est un facteur prédictif significatif de complications;
- Les hospitalisations prolongées augmentent le risque d'infection péristomiale;
- Une approche multidisciplinaire optimise la sécurité et les résultats de la GPE, les avantages et la facilité de pose des GPE ne doivent cependant pas conduire à porter des indications par excès.
